# Screening of Diatom Strains and Characterization of *Cyclotella cryptica* as A Potential Fucoxanthin Producer

**DOI:** 10.3390/md14070125

**Published:** 2016-07-08

**Authors:** Bingbing Guo, Bin Liu, Bo Yang, Peipei Sun, Xue Lu, Jin Liu, Feng Chen

**Affiliations:** 1 Institute for Food and Bioresource Engineering, College of Engineering, Peking University, Beijing 100871, China; guobb@pku.edu.cn (B.G.); caisanrenju@163.com (B.L.); ly_mikeyang@163.com (B.Y.); zixinsp@163.com (P.S.); gracexuelu@pku.edu.cn (X.L.); 2 South China Sea Bio-Resource Exploitation and Utilization Collaborative Innovation Center, Sun Yat-sen University, Guangzhou 510006, China

**Keywords:** fucoxanthin, *Cyclotella cryptica*, diatom, heterotrophic cultivation

## Abstract

Fucoxanthin has been receiving ever-increasing interest due to its broad health beneficial effects. Currently, seaweeds are the predominant source of natural fucoxanthin. However, the disappointingly low fucoxanthin content has impeded their use, driving the exploration of alternative fucoxanthin producers. In the present study, thirteen diatom strains were evaluated with respect to growth and fucoxanthin production potential. *Cyclotella cryptica* (CCMP 333), which grew well for fucoxanthin production under both photoautotrophic and heterotrophic growth conditions, was selected for further investigation. The supply of nitrate and light individually or in combination were all found to promote growth and fucoxanthin accumulation. When transferring heterotrophic cultures to light, fucoxanthin responded differentially to light intensities and was impaired by higher light intensity with a concomitant increase in diadinoxanthin and diatoxanthin, indicative of the modulation of Diadinoxanthin Cycle to cope with the light stress. Taken together, we, for the first time, performed the screening of diatom strains for fucoxanthin production potential and investigated in detail the effect of nutritional and environmental factors on *C. cryptica* growth and fucoxanthin accumulation. These results provide valuable implications into future engineering of *C. cryptica* culture parameters for improved fucoxanthin production and *C. cryptica* may emerge as a promising microalgal source of fucoxanthin.

## 1. Introduction

Fucoxanthin, a specific non-provitamin A carotenoid, is mainly found in brown seaweeds, diatoms and golden algae [1]. Fucoxanthin harbors a very unique structure with an allenic bond, a conjugated carbonyl and a 5,6-monoepoxide [2]. This structure endows fucoxanthin with a variety of biological activities, including anti-obesity and anti-diabetes [3,4,5,6], anti-cancer [7,8,9,10,11], anti-allergic [12,13], anti-inflammatory [14,15], anti-oxidation [14], and anti-osteoporotic activities [14]. Both in vitro and in vivo experiments indicated no toxicity of fucoxanthin [16,17,18].

Currently, fucoxanthin is mainly produced from seaweeds. Nevertheless, there are inherent shortcomings for seaweeds such as slow growth, low fucoxanthin content, insufficient yield, and quality concern due to the severe contamination of ocean by heavy metals [19,20,21,22,23,24]. By contrast, the marine algae diatoms, which grow rapidly, have high fucoxanthin content, and perform robustly in controlled bioreactors [25,26,27], are therefore considered as potential alternative producers of fucoxanthin. For example, the fucoxanthin content ranges from 0.224% to 2.167% of dry weight in diatoms, up to 100 times of that in seaweeds [28,29]. Moreover, some diatoms can grow in the dark using sugar as the only energy and carbon resource [30]. It has been reported that heterotrophic *Nitzschia laevis* grew well and reached high cell density [31], indicative of the potential of diatom fermentation for industrial production.

**Figure 1:**
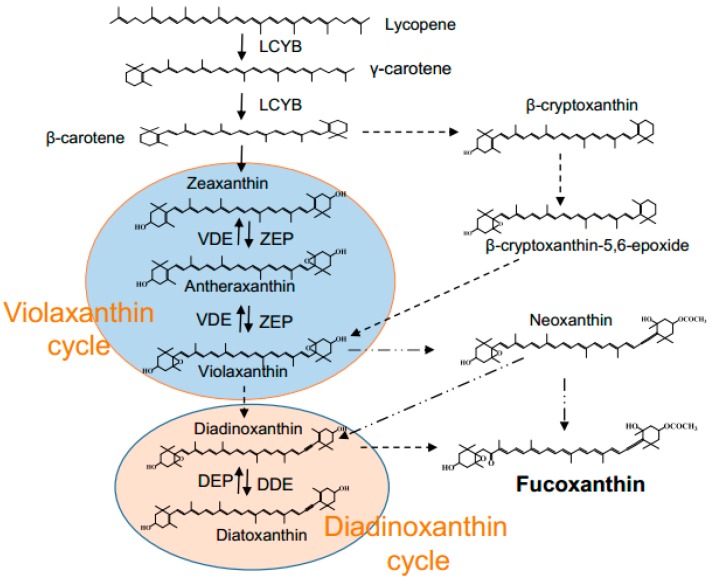
Proposed fucoxanthin biosynthetic steps in diatoms. LCYB: lycopene β-cyclase; VDE: violaxanthin de-epoxidases; ZEP: zeaxanthin epoxidases; DEP: diatoxanthin epoxidases; DDE: diadinoxanthin de-epoxidases. Solid arrows indicate steps that have been validated, while dotted arrows designate the steps to be defined.

The biosynthetic pathway of fucoxanthin remains unclear in diatoms [32,33]. It has been proposed that fucoxanthin can be either from neoxanthin or from diadinoxanthin (Figure 1). Violaxanthin is the precursor of neoxanthin, together with zeaxanthin and antheraxanthin constituting Violaxanthin Cycle. This cycle serves as a balanced cycle responding to a wide range of light intensity variation [32,34]. Upon high light, the algal cell tends to accumulate zeaxanthin at the expense of violaxanthin, leading to low level of neoxanthin and fucoxanthin as well. Diadinoxanthin Cycle, which consists of diadinoxanthin and diatoxanthin [35], serves the most important short-term photoprotective mechanism [36,37]. For example, when light intensity increases, diadinoxanthin tends to be converted to diatoxanthin for self-protection, leading to reduced fucoxanthin biosynthesis [38,39,40].

Growth and fucoxanthin content of diatoms may vary considerably across species and/or strains and are dependent on culture conditions. Therefore, it is imperative to employ a high performance diatom strain along with an optimal operational protocol for fucoxanthin production. There are only a limited number of reports about diatoms for fucoxanthin, with the focus on photoautotrophic growth and exploration of fucoxanthin extraction methods [2,28,41,42,43]. The utilization of heterotrophic growth of diatoms for fucoxanthin has been rarely touched [31,37,44]. The disappointingly low fucoxanthin production remains a large barrier for using diatoms as a potential source alternative to seaweeds. Comprehensive strain screening for fucoxanthin-rich producer and rational culture optimization represent feasible directions toward enhanced fucoxanthin production by diatoms, which to our best knowledge, have not been done. Hence, the main objective of the present study is to select a good strain capable of growing well both photoautotrophically and heterotrophically and optimize several key biological and engineering parameters for improved fucoxanthin production under laboratory conditions. To this end, we screened 13 naturally occurring diatom strains from several culture collection centers and identified a promising strain, *Cyclotella cryptica* (CCMP 333), with regard to growth and fucoxanthin production. This strain was investigated in detail in flasks to evaluate the effect of nutritional and environmental factors on growth and fucoxanthin accumulation. These results provide valuable implications into future exploration of *C. cryptica* for possible production of fucoxanthin.

## 2. Results

### 2.1. Fucoxanthin Production of Diatoms under Photoautotrophic Conditions

**Figure 2:**
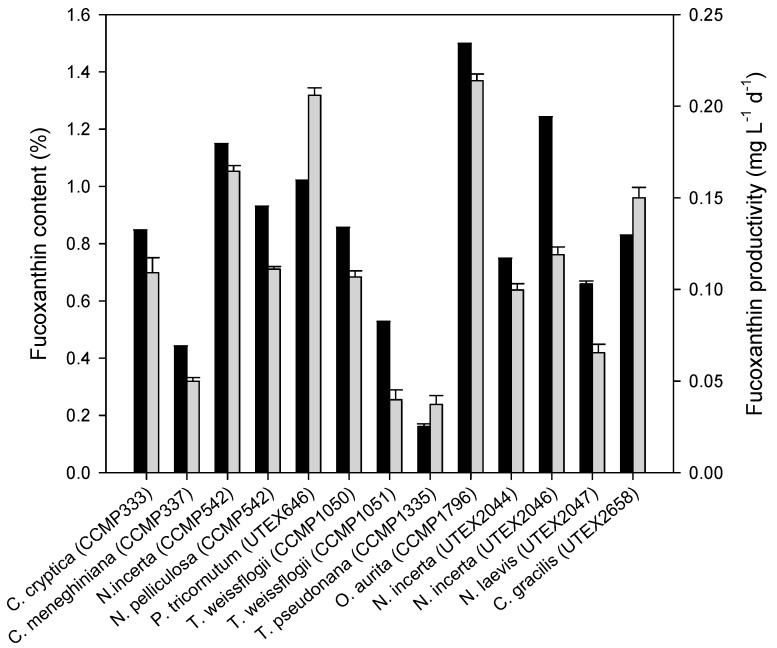
Fucoxanthin content and productivity of thirteen diatom strains under photoautotrophic conditions. Cells on Day 14 were harvested for analysis. Dark bars represent the fucoxanthin content and grey ones represent the fucoxanthin productivity. The fucoxanthin productivity was expressed as fucoxanthin yield divided by culture days. Data are means of three replicates and error bars indicate standard deviations.

The fucoxanthin production potential of thirteen diatoms was first evaluated under photoautotrophic conditions with the light intensity of 30 μmol·m^−2^·s^−1^. As indicated by Figure 2, most of the strains tested had a fucoxanthin content of over 0.8% of dry weight, and *O. aurita* was the strain with highest fucoxanthin content (1.50%). By contrast, *Thalassiosira pseudonana* (CCMP 1335) exhibited the lowest fucoxanthin content (below 0.2%). As for fucoxanthin productivity, nine of the thirteen strains had a value above 0.1 mg·L^−1^·d^−1^, with *Odontella aurita* (CCMP 1796) and *Phaeodactylum* *tricornutum* (UTEX 646) being the greatest. By contrast, *Thalassiosira weissflogii* (CCMP 1051) and *T. pseudonana* had the lowest fucoxanthin productivity, which were less than 0.05 mg·L^−1^·d^−1^. 

### 2.2. Growth and Fucoxanthin Production Potential of Diatoms under Heterotrophic Conditions

**Figure 3:**
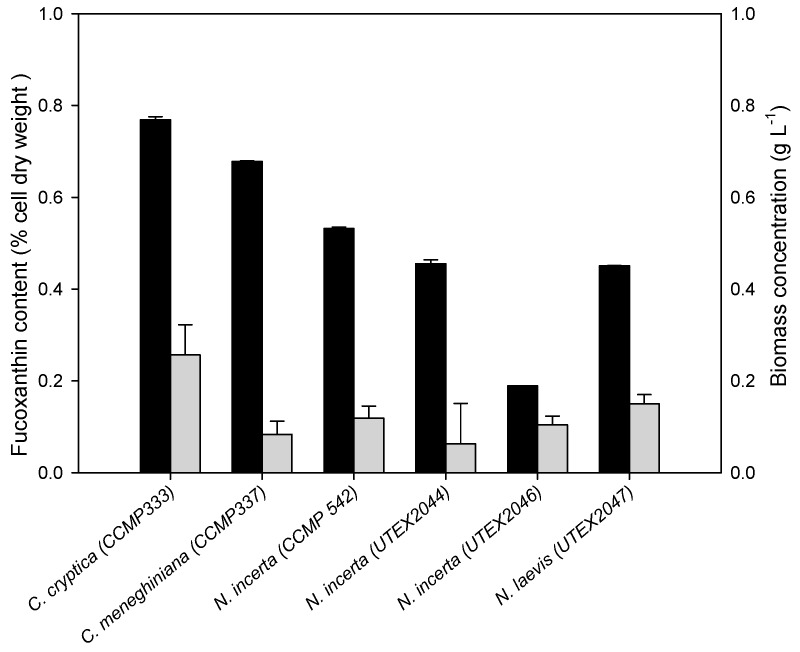
Fucoxanthin content (**black** column) and biomass concentration (**gray** column) of diatom strains capable of growing on glucose without light. Cells on Day 6 were collected for analysis. The solid black bar and grey bar represent the fucoxanthin content and biomass concentration, respectively. Data are means of three replicates and error bars indicate standard deviations.

Of the thirteen diatoms tested, only six could grow on glucose without light, namely, *Cyclotella cryptica* (CCMP 333), *Cyclotella meneghiniana*, *Navicula incerta* (CCMP 542), *Navicula incerta* (UTEX 2044), *Navicular incerta* (UTEX 2046) and *Nitzschia laevis* (UTEX 2047), though the biomass concentration achieved was relatively low (Figure 3). Comparatively, *C. cryptica* had a higher biomass concentration than the other five strains. In addition, *C. cryptica* had the highest fucoxanthin content, which was 0.77% of dry weight and comparable to that under photoautotrophic conditions. Interestingly, *C. meneghiniana* had a higher fucoxanthin content under heterotrophic growth conditions than under photoautotrophic conditions. On the contrary, the other four strains showed a decrease in fucoxanthin content under heterotrophic growth conditions compared to under photoautotrophic conditions. Taken together, *C. cryptica* grew relatively better among the six strains under heterotrophic growth conditions and its fucoxanthin content was comparable to that of other diatom strains under photoautotrophic conditions, thus it was chosen for the subsequent investigation. 

### 2.3. Effect of Nitrate and/or Light Supply on Growth and Fucoxanthin Production of C. cryptica

**Figure 4:**
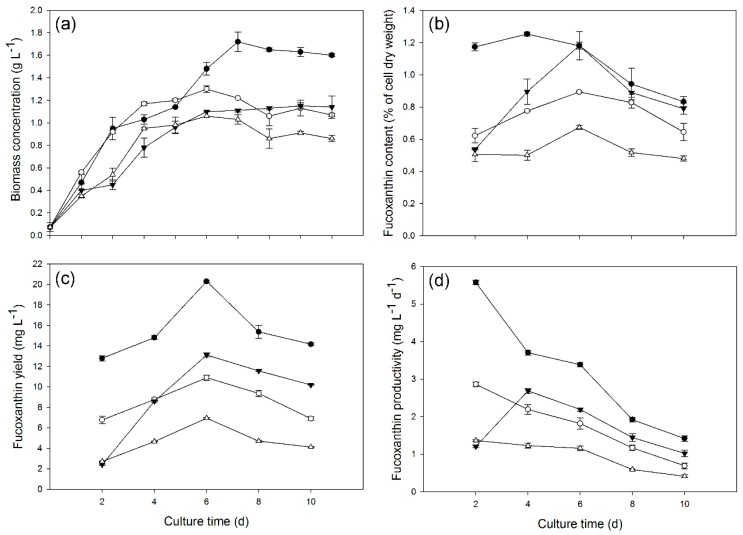
Biomass concentration (**a**); Fucoxanthin contents (**b**); Fucoxanthin yield (**c**); and Fucoxanthin productivity (**d**) of *C. cryptica*. Solid circle and empty circle represent the culture with or without nitrate in the light, and solid triangle down and empty triangle up represent the culture with or without nitrate under dark, respectively. The nitrate concentration is 1.00 g·L^−1^ and the light intensity is 30 μmol·m^−2^·s^−1^. Data are means of three replicates and error bars indicate standard deviations.

We found that *C. cryptica* grew better in modified SK medium than in F/2 medium under heterotrophic conditions (data not shown). Based on the modified SK medium, we further tested the effect of nitrate and/or light supply on growth and fucoxanthin production of *C. cryptica* (Figure 4a,b). Nitrate supply showed almost no effect on *C. cryptica* growth before entering the stationary phase (Day 6); afterwards, the algal cells showed a decline in biomass concentration for the control cultures while a slight increase for the nitrate-supplied cultures (Figure 4a). Consequently, the final biomass concentration reached 1.15 g·L^−1^ for the nitrate-supplied cultures, 26.4% higher than the control. By contrast, light supply had a more significantly positive effect on cell growth and led to a greater biomass concentration than the control during the whole culture period. Noteworthy, similar to the control, the light-supplied cells reached the highest biomass concentration (1.30 g·L^−1^) on Day 5, followed by a gradual decline. The supply of both nitrate and light further promoted the cell growth and biomass concentration reached the maximum of 1.72 g·L^−1^ on Day 6, which was 55.0% higher than that of the control cultures.

The effect of nitrate and/or light supply on fucoxanthin content is shown in Figure 4b. The control cultures maintained a relatively stable fucoxanthin content. Nitrate supply benefited the accumulation of fucoxanthin, which increased rapidly after Day 2 and reached the maximum of 1.18% on Day 6, 75.4% higher than that of the control. Light supply had a similar effect to nitrate supply and promoted fucoxanthin accumulation, with the maximum content of 0.89% of dry weight achieved on Day 6. By contrast, the supply of both nitrate and light promoted fucoxanthin accumulation more significantly, which occurred on Day 2 and then increased slightly to the maximum content of 1.29% of dry weight on Day 4, 86.0% higher than the maximum of control on Day 6. Interestingly, under all tested conditions, fucoxanthin content increased as the cells grew and started to decline once entering the stationary growth phase, indicating that fucoxanthin accumulation is possibly growth dependent. 

Both nitrate and light supply independently promoted fucoxanthin yield considerably (Figure 4c). The combination of nitrate and light supply further increased fucoxanthin yield. Regardless of the culture conditions, the maximum fucoxanthin yield was achieved on Day 6. Specifically, the maximum fucoxanthin yield under the supply of both nitrate and light was 20.30 mg·L^−1^, 192.5% higher than that of the control. As for the productivity, the supply of nitrate and light individually or in combination all promoted it, with the combination of nitrate and light being most effective (Figure 4d). Clearly, fucoxanthin productivity remained relatively high in early growth days and declined gradually, regardless of culture conditions. This may indicate that *C. cryptica* in exponential growth phase is preferred for fucoxanthin production. 

### 2.4. Effect of Light Intensity on Growth and Fucoxanthin Production

**Figure 5:**
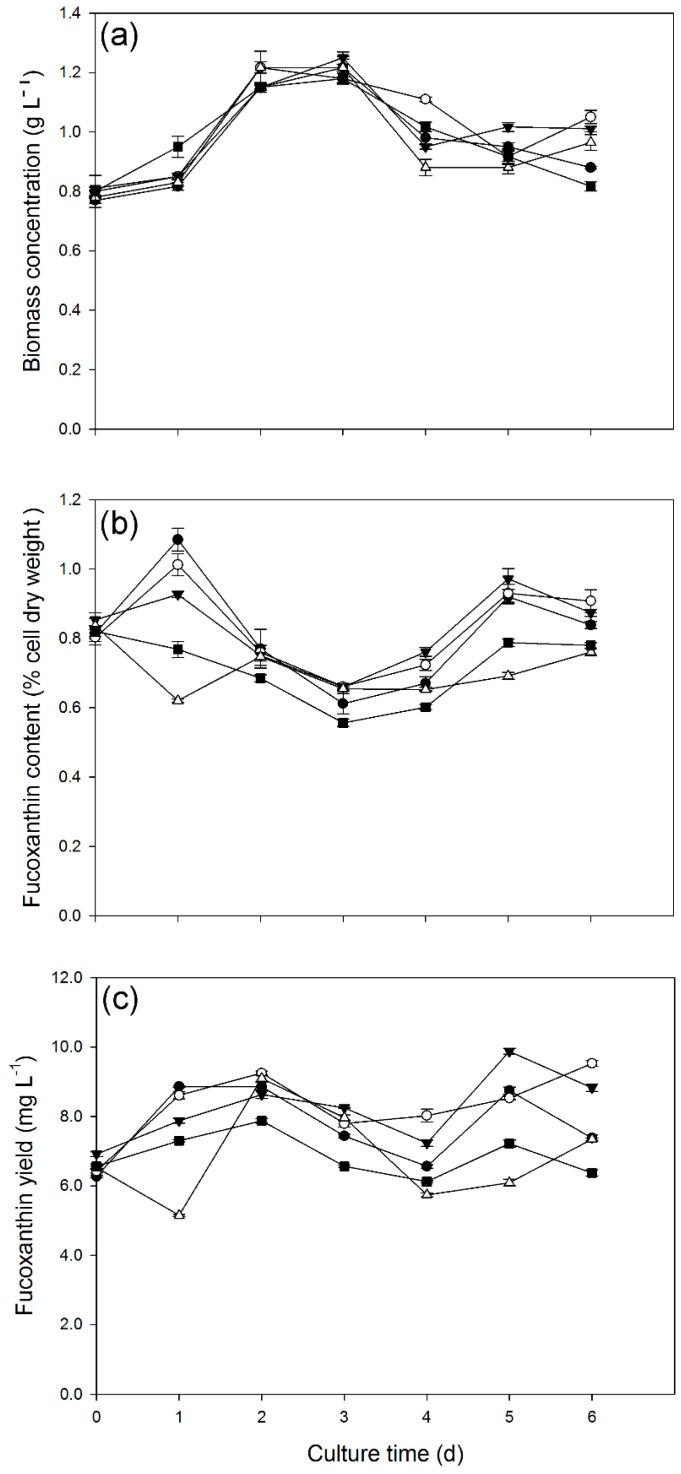
Biomass concentration (**a**); Fucoxanthin contents (**b**); and Fucoxanthin yield (**c**) under different light intensity. Solid circle, empty cycle, solid triangle down and empty triangle up represent an illumination intensity condition of 10, 20, 30, and 40 μmol·m^−2^·s^−1^, respectively, with solid square as the dark-growing control. Data are means of three replicates and error bars indicate standard deviations.

We demonstrated that light supply affected the growth and fucoxanthin production of *C. cryptica*. To further investigate the effect of light intensities, four light intensities of 10, 20, 30, and 40 μmol·m^−2^·s^−1^ were applied (Figure 5). The *C. cryptica* cells were first grown without light and then subjected to different light intensities, using the dark-grown cells as the control. Obviously, light intensity had no significant effect on cell growth under this tested conditions (Figure 5a). The cells began to decline after three days of cultivation (Figure 5a), probably as a result of nutrition depletion. By contrast, light intensities influenced the cellular fucoxanthin content differentially: light intensities between 10 and 30 μmol·m^−2^·s^−1^ promoted the fucoxanthin accumulation while 40 μmol·m^−2^·s^−1^ had no beneficial effect (Figure 5b). Quantitatively, on Day 1, the fucoxanthin content was 1.08% of dry weight under the light intensity of 10 μmol·m^−2^·s^−1^, 33.3% higher than of the control (0.76%), while the fucoxanthin content under the light intensity of 40 μmol·m^−2^·s^−1^ was 0.62%, lower than that of the control. The effect of light intensity on fucoxanthin yield was also examined (Figure 5c). Overall, low light intensities of 10–30 μmol·m^−2^·s^−1^ were better than the relatively high light intensity of 40 μmol·m^−2^·s^−1^. 

### 2.5. Effect of Light Intensity on Diadinoxanthin and Diatoxanthin

**Figure 6:**
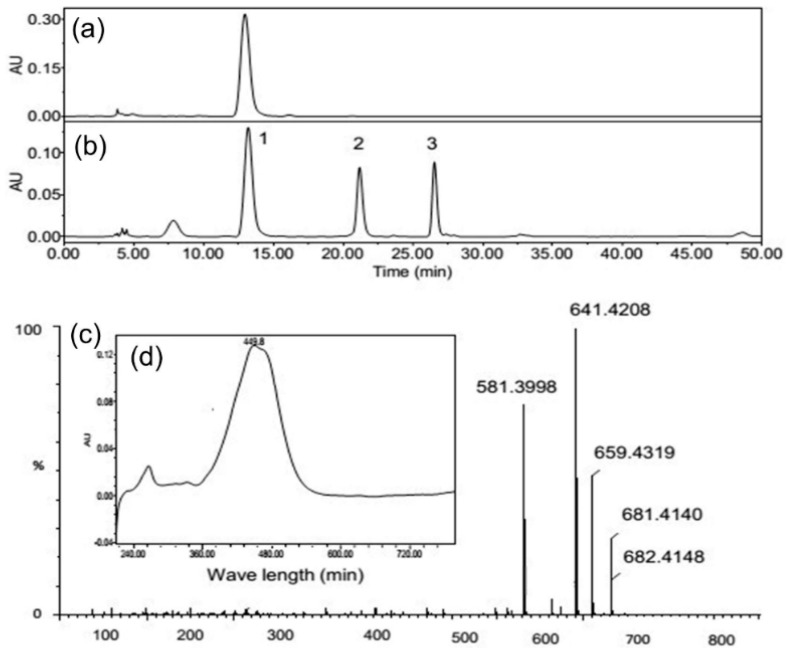
HPLC (High Performance Liquid Chromatography) chromatograms of fucoxanthin standard (**a**) and the crude extract of *C. cryptica* (**b**). Mass spectroscopy (**c**) of peak 1 in (**b**) and its visible absorption spectra (**d**). Absorbance in (**a**) and (**b**) was recorded at 449 nm. Peaks were identified as follows: 1, fucoxanthin; 2, diadinoxanthin; and 3, diatoxanthin.

HPLC analysis of the carotenoid composition of *C. cryptica* extracts found two unknown major peaks right after fucoxanthin (Figure 6). Fucoxanthin was identified by comparing the retention time of a fucoxanthin standard (Figure 6a,b), and was further confirmed by its mass spectrometry (Figure 6c) and UV-visible spectrum (λ_max_ at 449.8 nm) (Figure 6d). To identify unknown peaks 2 and 3, mass spectrometry analysis was performed. Through the comparison of their fragment patterns with previous reports [45,46], they were annotated to be diadinoxanthin and diatoxanthin, respectively (Table 1).

**Figure 7:**
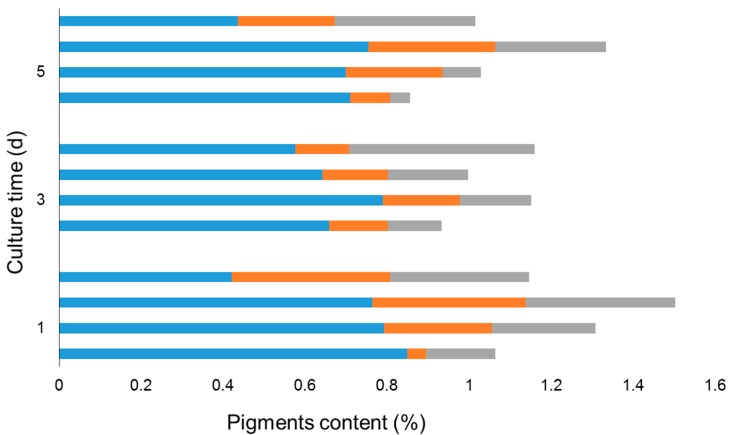
Fucoxanthin content (**blue** bar), Diadinoxanthin content (**orange** bar) and Diatoxanthin content (**grey** bar) under different light intensity. Bars from the bottom up in every group represent the light intensity of 10, 20, 30, and 40 μmol·m^−2^·s^−1^, respectively. Diadinoxanthin and diatoxanthin contents were quantified as fucoxanthin equivalents with the same method.

Clearly, as the light intensity increased from 10 to 30 μmol·m^−2^·s^−1^, diadinoxanthin and diatoxanthin increased while fucoxanthin declined on Day 1 (Figure 7). When the light intensity further reached to 40 μmol·m^−2^·s^−1^, diadinoxanthin and diatoxanthin remained relatively stable; by contrast, fucoxanthin dropped sharply. This similar pattern was also observed on Day 3 and Day 5.

## 3. Discussion

The growth and fucoxanthin synthesis of diatoms have been previously reported under photoautotrophic conditions [2,42]. Heterotrophic cultivation of diatoms has also been documented, but not for fucoxanthin production [47,48]. In the present study, we for the first time performed a screening study using thirteen diatom strains across ten species, with respect to their growth and fucoxanthin accumulation under both photoautotrophic and heterotrophic conditions (Figure 2 and Figure 3). *C. cryptica* (CCMP 333) was selected because it could grow heterotrophically on glucose better and accumulated more fucoxanthin than other tested diatom strains (Figure 3). Sugars, glucose in particular, are the commonly used organic carbon source for the fermentation of microorganisms including algae [31,49,50,51]. Algae fermentation has some significant advantages and thus has been considered as a promising production strategy for value-added products [31,49,52]. Nevertheless, the biomass concentration achieved in our study was relatively low (Figure 3). We replaced F/2 medium with modified SK medium, which was derived from Pahl et al. [37] with minor modifications, and observed a considerable increase in biomass concentration (Figure 2, Figure 3 and Figure 4).

Nitrogen is a primary nutrient for algae growth. There are multiple nitrogen sources such as organic nitrogen from tryptone, yeast extract and amino acids, and inorganic nitrogen from nitrate, ammonium and urea. The preference of algae for nitrogen sources is species and/or strain dependent [53,54,55,56,57,58]. The modified SK medium used in the present study contains the organic nitrogen of tryptone and yeast extract. We found that nitrate supply to modified SK medium benefited *C. cryptica* growth leading to a higher biomass concentration (Figure 4). This was in line with previous studies that high level of available nitrogen promoted biomass accumulation of diatoms [20] as well as of other algae strains [57,58]. Interestingly, the higher biomass concentration caused by nitrate supply only occurred in the stationary growth phase (Figure 4a), which may indicate that the nitrogen level of modified SK medium is not sufficient for *C. cryptica* growth under our tested conditions. Nitrogen also plays an important role in regulating cellular metabolites. Nitrate needs to be converted to nitrite and then to ammonium for assimilation by algae. Ammonium is used to synthesize glutamate, which is a precursor for the biosynthesis of chlorophyll for photosynthesis process [28]. Nitrate supply might upregulate the biosynthesis of chlorophyll and, consequently, promote fucoxanthin accumulation (Figure 4b), as fucoxanthin is a core part of photosystem in diatoms. Consistent with this, downregulation of glutamate was observed upon nitrogen deprivation, accompanied by a decrease in chlorophyll and fucoxanthin contents [59].

Light has dual effects on fucoxanthin biosynthesis: light benefits fucoxanthin accumulation when in low intensities, but impairs fucoxanthin when intensity exceeds a certain value, say 40 μmol·m^−2^·s^−1^ in our study (Figure 5), in line with the previous reports that algal fucoxanthin content was higher under low light intensity [60,61]. Thus, higher fucoxanthin production was obtained under low light intensities (Figure 5c). The beneficial effect of low light intensity on fucoxanthin content and production was also observed in previous reports [33,42]. We hypothesize that the fucoxanthin variation caused by light may be related to the modulation of Diadinoxanthin Cycle [36,37]. Under high light intensities, to cope with the abiotic stress, diatom cells tend to convert diadinoxanthin to diatoxanthin at the expense of fucoxanthin, leading to reduced fucoxanthin biosynthesis (Figure 1).

In addition to nitrogen and light, there are many other factors worth of investigation for growth and fucoxanthin production of *C. cryptica*, including different medium and agitation conditions [62]. Our study highlights the production potential of *C. cryptica*, but its biomass concentration is still relatively low. Nevertheless, our study represents a pioneering effect of using heterotrophic diatom cultures for fucoxanthin. The future exploration of this alga for fucoxanthin production may lie in the optimization of heterotrophic parameters for improving the glucose-to-biomass conversion efficiency and cell density and the development of rational illumination of heterotrophic cultures for maintaining fucoxanthin content.

## 4. Materials and Methods

### 4.1. Strains and Culture Conditions

Thirteen diatom strains were used, including *C. cryptica* (CCMP 333), *C. meneghiniana* (CCMP 337), *N. incerta* (CCMP 542), *N. pelliculosa* (CCMP 543), *T. weissflogii* (CCMP 1050), *T. weissflogii* (CCMP 1051), *T. pseudonana* (CCMP 1335), *O. aurita* (CCMP 1796), *P. tricornutum* (UTEX 646), *N. incerta* (UTEX 2044), *N. incerta* (UTEX 2046), *N. laevis* (UTEX 2047), and *Ch. gracilis* (UTEX 2658). They were obtained from National Center for Marine Algae and Microbiota (NCMA) or Culture Collection of Algae at The University of Texas at Austin (UTEX).

For the screening experiment, all these strains were cultivated in 250 mL Erlenmeyer flasks containing 50 mL of sterile F/2 medium (recipe from NCMA) under an illumination intensity of 30 μmol·m^−2^·s^−1^ till the exponential stage. Alga cells were then inoculated at 10% (*v*/*v*) into 1000 mL conical flasks containing 400 mL of the same medium with a salinity of 27.5‰ and grown at 25 °C for 14 days. For photoautotrophic cultures, the illumination intensity was 30 μmol·m^−2^·s^−1^ with a light/dark regime of 12 h/12 h. For heterotrophic cultures, ten grams of glucose were added per liter of medium. All medium was sterilized at 121 °C for 20 min. Alga cells were harvested through centrifugation and then freeze-dried for 48 h before being used to extract pigments.

For the growing experiment of *C. cryptica*, modified SK medium was used with modifications reported by Pahl et al. [54]. The base medium consists of (per liter) 22.5 g sea salt (Sigma, St. Louis, MO, USA), 0.66 g MgSO_4_·7H_2_O, 1.6 g tryptone, 0.8 g yeast extract, 50.5 mg KH_2_PO_4_, 34 mg H_3_BO_3_, 20 mg FeSO_4_·7H_2_O, 4.3 mg MnCl_2_·4H_2_O, 0.3 mg ZnCl_2_, 0.13 mg CoCl_2_·6H_2_O and 0.03 mg Na_2_MoO_4_·2H_2_O, and was supplemented with 10 g·L^−1^ glucose and 480 mg·L^−1^ Na_2_SiO_3_·5H_2_O. The pH of the medium was adjusted to 7.5 prior to autoclaving at 121 °C for 20 min. 

For the nitrate-induced experiment of *C. cryptica*, 1.00 g·L^−1^ sodium nitrate was added in the base medium before sterilization, while base medium without supplement of sodium nitrate was used as the control. Both of the two groups were cultured in continuous light (30 μmol·m^−2^·s^−1^) and dark conditions with an orbital shaking of 150 rpm at 25 °C, respectively. For the dark-light experiment, the culture was cultivated for 4 days to reach the exponential phase in dark and then exposed to different continuous light intensities, 10, 20, 30, or 40 μmol·m^−2^·s^−1^.

### 4.2. Determination of Biomass Concentration and Fucoxanthin Production

Samples were measured and the biomass concentration was calculated as previously described [63]. Briefly, five milliliters of cell suspension were sampled to determine the biomass concentration. Cells were washed for two times with distilled water before being filtered on a pre-weighted Whatman GF/C filter (Cat No. 1822-047). The filters containing the algae were dried in a vacuum oven at 80 °C for 24 h and was subsequently cooled down to room temperature for weighing. The biomass concentration is expressed as cells dry weight per liter.

The maximum specific growth rate (μ_max_) was calculated according to:

μ_max_ = (ln*X*_2_ − ln*X*_1_)/(*t*_2_ − *t*_1_) (1)

where *X*_2_ and *X*_1_ are the dry weight (g·L^−1^) at time *t*_2_ and *t*_1_, respectively.

The fucoxanthin yield was the result of fucoxanthin content multiplied by biomass concentration, while the fucoxanthin productivity was expressed as fucoxanthin yield divided by culture days.

### 4.3. Pigments Extraction

For all extraction procedures, 20 mg of lyophilized cell powder was ground and then extracted with 3 mL analytical ethanol under dim light. After shaking for 1 h at room temperature, the whole solutions carrying grinded cells were centrifuged at 3000 r/min for 5 min. The supernatant was collected and the pellets were used to extract pigments for another two times under the same conditions. All three supernatants were combined and evaporated with N_2_ in dim light. Subsequently, crude pigments were dissolved in pure ethanol and filtered through a 0.22 μm filter (Anpel, Shanghai, China) prior to HPLC and LC-MS analysis.

### 4.4. Analysis of Pigments by HPLC and LC-MS

Fucoxanthin was detected using Waters 2695 HPLC system (Waters, Milford, MA, USA) with a PDA detector and a C_18_ reverse phase bar (5 mm particle size, 250 mm × 4.6 mm ID). Specific analysis method was set according to Kim et al. [2] with minor modifications. Briefly, the mobile phase consisted of methanol and water with a flow rate of 0.8 mL·min^−1^. In the gradient condition, methanol/water ratio was increased from 90:10 to 100:0 in 30 min and then the 100% methanol flow held for another 20 min. The chromatogram was recorded at 449 nm. The fucoxanthin standards were used for the construction of calibration curve at a concentration range of 10–250 μg·mL^−1^. Pigments were also confirmed by liquid chromatography-mass spectrometry (LC-MS, Waters Xevo G2QTOF, Milford, MA, USA) with an atmospheric pressure chemical ionization (APCI) using the same chromatographic column under the same mobile phase conditions.

### 4.5. Statistical Analysis

All cultivations were performed at least in duplicate. All determinative data were collected from triplicate samples and the final values were expressed as mean value ± standard deviation. Statistical significance was evaluated by ANOVA and *t*-test using SPSS programs (Version 19.0, IBM SPSS, Chicago, IL, USA) at a level of *P* < 0.05.

## 5. Conclusions

In summary, we for the first time screened thirteen diatom strains with respect to their growth and fucoxanthin accumulation, and selected *C. cryptica* as a promising producer for fucoxanthin. Detailed investigation revealed that the supply of nitrate and light individually or in combination promoted growth and fucoxanthin accumulation of *C. cryptica*. When transferring heterotrophic algal cultures to light, fucoxanthin responded differentially to light intensities by regulating the Diadinoxanthin Cycle and was upregulated by low intensities. Our study represents a research starting point and provides valuable implications into future engineering of culture parameters for improved fucoxanthin production by *C. cryptica*.

## Figures and Tables

**Table 1:** Compounds tentatively assigned in the crude extracts of *C. cryptica*.

Peak No.	Compound	t_r_ * (min)	λ_max_ (nm)	Ionization Type	Mass Spectra M^+^ *m*/*z* (Relative Intensity, %)
2	Diadinoxanthin	21.059	444.9 475.3	[M+H]^+^ [M+H-H_2_O]^+^	565.4(10); 583.4(100); 584.4(50); 585.4(10)
3	Diatoxanthin	26.257	449.8 478.9	[M+H]^+^ [M+H-H_2_O]^+^	549.4(10); 567.4(100); 568.4(50); 569.4(10)

t_r_ *: retention time.
